# SWAU-Net: Longitudinal Prediction of Geographic Atrophy via Sliding-Window Attention

**DOI:** 10.3390/life16020303

**Published:** 2026-02-10

**Authors:** Peter Racioppo, Ziyuan Chris Wang, SriniVas R. Sadda, Zhihong Jewel Hu

**Affiliations:** 1 Doheny Image Analysis Laboratory, Doheny Eye Institute, 150 North Orange Grove Blvd, Pasadena, CA 91103, USA; pracioppo@doheny.org (P.R.); zwang@doheny.org (Z.C.W.); 2 Department of Ophthalmology, University of California, Los Angeles, CA 90095, USA; ssadda@doheny.org

**Keywords:** geographic atrophy, longitudinal prediction, regularized transformer, low-data regime

## Abstract

Age-related macular degeneration (AMD) is the leading cause of central vision loss in aging populations. Geographic atrophy (GA) is the advanced, non-neovascular form of AMD. Predicting the longitudinal progression of GA remains a critical challenge in ophthalmic clinical practice and clinical trial design. Forecasting the trajectory of GA is complicated by highly variable growth rates and the inherent scarcity of long-term, high-quality imaging data. To address these challenges, we introduce the Sliding Window Attention U-Net (SWAU-Net), a hybrid architecture that integrates Transformer-based temporal modeling of GA growth with precise spatial modeling of GA location with a U-Net convolutional neural network (CNN). To ensure generalization in the low-data regime, SWAU-Net embeds explicit temporal and geometric consistency priors via a weight-shared Sliding Window Attention core and feature-level regularization that preserves sparse, high-frequency lesion boundaries across frames. Experimental results demonstrate that these structural constraints prevent the model from overfitting to imaging noise, achieving a Growth Mask Dice Similarity Coefficient (DSC) of 0.66 (representing the spatial overlap between the predicted and ground truth lesion expansion regions), a significant improvement over unregularized Transformer and standard recurrent baseline models. Our framework provides a robust tool for predicting GA lesion trajectories, potentially supporting more efficient clinical trial designs and personalized patient monitoring.

## 1. Introduction

### 1.1. Geographic Atrophy and Retinal Imaging

Geographic Atrophy (GA) is the advanced, non-neovascular form of age-related macular degeneration (AMD), representing a leading cause of irreversible central vision loss among elderly populations [1,2]. GA arises from progressive degeneration of the retinal pigment epithelium (RPE), photoreceptors, and the underlying choriocapillaris, producing sharply demarcated, map-like regions of atrophy in the macula. While visual acuity is often preserved until the fovea is directly involved, the presence of parafoveal scotomas can lead to a profound decline in functional vision. These dense scotomas can interfere with high-acuity visual tasks such as reading and face recognition by fragmenting the visual field and reducing contrast sensitivity [3,4]. Direct involvement of the fovea marks the transition to a terminal loss of central visual acuity.

GA is estimated to affect over 5 million people worldwide, which is expected to rise as global populations age [5,6]. In a large prospective natural history study, the median enlargement rate was found to be 2.1 mm^2^/year, though individual rates vary widely depending on baseline lesion size and prior growth history [7].

Quantitative characterization of GA progression has become a key endpoint in both natural-history studies and interventional trials [8]. Critical prognostic features include junctional zone hyperautofluorescence, drusen regression, hyperreflective foci, and choroidal thinning—reflecting local RPE and photoreceptor stress that anticipates lesion expansion [9,10].

Fundus Autofluorescence (FAF) remains the gold-standard non-invasive imaging modality for monitoring GA. FAF visualizes lipofuscin accumulation and loss within the RPE, offering high-contrast delineation of atrophic borders [11]. Complementary to FAF, Optical Coherence Tomography (OCT) provides volumetric cross-sectional views that resolve structural biomarkers [12]. Longitudinal FAF and OCT imaging together enable clinicians to measure both the spatial extent and evolution of GA lesions, supporting visual-function prediction and treatment evaluation in clinical trials [13].

Forecasting GA progression—particularly from limited historical data—remains a critical unmet need for personalizing monitoring schedules and therapeutic decision-making. This challenge arises because GA expansion is a heterogeneous process driven by the local retinal microenvironment and baseline lesion geometry [8,14]. Progression is rarely uniform; instead, it often manifests through the sudden coalescing of satellite lesions or irregular protrusions into healthy tissue. Because a single clinical snapshot cannot capture this underlying process, accurate forecasting requires models that can interpret the subtle, time-varying shifts at the junctional zone, where metabolic stress precedes visible structural collapse.

Furthermore, the rate of GA expansion is highly dependent on the phenotype of junctional zone Fundus Autofluorescence (FAF). Clinical studies have categorized these into specific patterns—including focal, banded, patchy, and diffuse—with banded and diffuse patterns typically associated with significantly faster progression [14,15]. In this study, we utilized a deep learning approach to extract these high-dimensional features implicitly from the raw FAF and GA masks rather than using manual categorical labeling.

**Figure 1:**
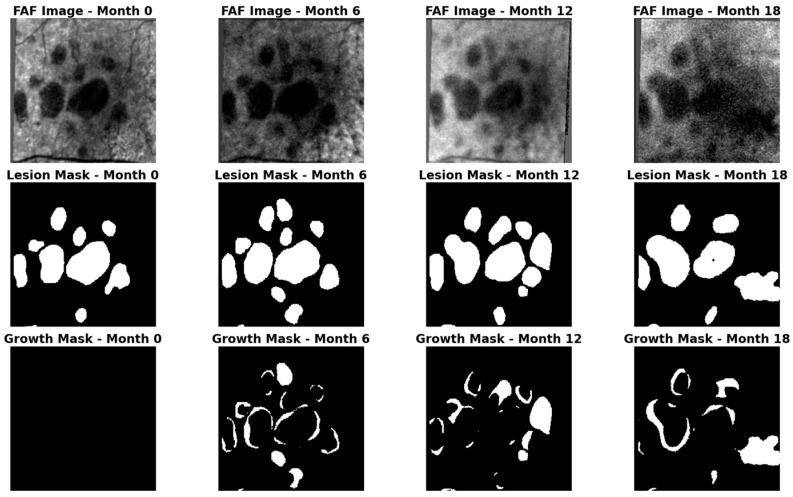
(**Top**) FAF scan, with geographic atrophy (GA) clearly visible as the dark central region; (**Center**) Mask of the GA region, annotated and certified by expert graders at the Doheny Image Reading Center; (**Bottom**) Mask of the growth region since the previous scan (calculated as the pixel-wise difference between adjacent longitudinal GA masks, i.e., $Growth\;Mask_{i}=Lesion\;Mask_{i}-Lesion\;Mask_{i-1}$, representing the new area of atrophy within the 6-month interval).

The spatial and temporal complexity of the lesion’s “growth front” is exemplified in Figure 1, which depicts a representative sequence (one of 66 in this study) of GA progression over 18 months along with a human-annotated mask of the GA region, and the resulting mask of the growth region. This sequence highlights the sparse, irregular nature of the expansion regions typical of the disease, which necessitates high-fidelity spatiotemporal modeling.

### 1.2. Spatiotemporal Deep Learning

Early frameworks such as ConvLSTMs and 3D ConvNets [16,17] established spatiotemporal encoder–-decoder architectures for forecasting longitudinal image sequences and video data. These were later improved by attention-based models, such as the Vision Transformer (ViT), which are better at capturing long-range spatial relationships across an image [18,19].

While Transformer-based architectures achieve high performance in general computer vision, they typically require massive datasets to generalize effectively. In medical imaging—where longitudinal data is often scarce—directly training these models can lead to overfitting, where the model memorizes noise rather than learning biological trends. To address this, hybrid designs have become standard; architectures such as TransUNet, MedT, and UTNet combine the local reliability of convolutions with the broader reasoning of Transformers to maintain accuracy in low-data settings [20,21,22].

A particularly effective branch of this research leverages hierarchical window-based attention, as seen in the Swin Transformer [23] and its medical adaptation, Swin-UNet [24], which allow the model to build a global understanding of an image from local patches. Other recent advancements, such as UniFormer [25] and Multiscale Vision Transformers [26], further refine this by extracting features at multiple scales to better capture complex, time-varying changes in anatomy.

### 1.3. Deep Learning for GA Detection and Forecasting

Recent deep learning approaches have achieved expert-level segmentation and detection of GA lesions across FAF and OCT modalities [27,28,29,30,31]. Building upon foundational work in automated GA segmentation using deep convolutional and deconvolutional neural networks [32,33,34], subsequent research has leveraged self-attention architectures to enhance feature discovery across AMD and Stargardt disease [35]. These multi-modal approaches have further evolved to resolve structural biomarkers across both SD-OCT and FAF imaging [36]. The research focus is now shifting from static segmentation to temporal forecasting. Prior works have studied applications of CNN–recurrent neural network (RNN) hybrids [37,38,39] to model temporal dependencies across successive imaging visits.

A limitation of standard CNN-RNN models is that they often compromise detail by collapsing 2D images into 1D vectors to process time. In contrast, more effective spatiotemporal models maintain the integrity of the retinal anatomy by processing spatial dimensions and temporal changes simultaneously [16]. Furthermore, FAF images and GA masks have different noise profiles. To prevent signal interference, hybrid encoders have been shown to improve accuracy by processing these distinct data types separately before merging them [40]. Because regions of growth are typically thin, irregular expansion bands, conventional model layers tend to over-smooth these regions. Gated or attention-modulated blocks have proven effective at preserving the sharp edge contrast and small-scale structural detail necessary to track the growth front [41,42,43].

Unlike CNN–RNN pipelines, attention mechanisms maintain a broader view of both space and time, avoiding the vanishing gradient issues common in older recurrent models [44]. However, while recurrent networks enforce steady growth through a fixed mathematical transition, unconstrained attention mechanisms lack a built-in understanding of time. In low-data settings, this high flexibility can lead to overfitting, where the model memorizes imaging artifacts rather than learning meaningful biological trends [45].

### 1.4. Main Contributions of Our Deep Learning Architecture

To address the challenges of using CNN–RNNs and Transformers alone, we introduce the Sliding Window Attention U-Net (SWAU-Net), a hybrid architecture that incorporates structural and temporal “priors” to stabilize GA forecasting. SWAU-Net is designed to remain robust in the low-data regime through three key principles:

- A regularized U-Net for spatial detail: To resolve the thin, irregular structure of GA growth regions, the model’s backbone is constrained to preserve boundary detail while filtering out noise. Refined residual blocks prevent the over-smoothing of junctional zone features, ensuring the growth front remains sharp even when training data is limited [46].
- Sliding Window Attention (SWA): To ensure the model generalizes across time, we enforce a “temporal stationarity” prior through architectural weight-sharing. By applying the same attention parameters across shifted windows, we create a structural bottleneck that prevents the model from memorizing specific visits. Instead, it is forced to learn a generalized, time-invariant transition function—effectively capturing the underlying biological “velocity” of GA expansion across the retina.
- Decoupled Dynamics Network (DynNet): We physically separate the task of identifying the current disease (state estimation) from the task of predicting future changes (evolution). By decoupling these functions, the encoder and SWA core can focus on producing a stable map of the atrophy, while a separate module (DynNet) is dedicated purely to modeling how those features evolve over time.

### 1.5. Study Objectives

The primary objective of this work is to develop and validate a robust deep-learning framework, SWAU-Net, for the longitudinal forecasting of geographic atrophy expansion. Given the high variability of GA growth and the scarcity of long-term imaging data, this model was designed to prioritize structural and temporal stability over raw parameter count. We aim to validate whether a regularized, hybrid CNN-Transformer architecture can outperform standard recurrent and unconstrained attention models in predicting the sparse growth frontiers of GA within a mid-sized clinical cohort.

## 2. Materials and Methods

### 2.1. Data

The GA dataset consists of deidentified longitudinal imaging data of 66 eyes from 66 patients (aged 60 years or older; both sexes), obtained from the Doheny Image Reading and Research Lab (DIRRL) database, with FAF imaging (Spectralis HRA + OCT 1.11.2.0, Heidelberg Engineering, Heidelberg, Germany) performed at the initial baseline visit—representing the study start point for each patient—and at six-, twelve-, and eighteen-month follow-ups. Inclusion criteria for this study required participants to have clear ocular media, adequate dilation and fixation for high-quality imaging, and GA lesions fully contained within the FAF field with adjacent banded or diffuse hyperautofluorescence. Eligible eyes were required to have a total GA lesion size between 1.25–17.5 mm^2^ and a Best Corrected Visual Acuity (BCVA) between 19 and 48 ETDRS letters. Exclusion criteria included evidence of choroidal neovascularization (CNV) or other ocular diseases and atrophies not related to AMD.

Based on established natural history data for geographic atrophy, a sample size of 38 eyes is required to detect a 25% difference in enlargement rates with 80% power at a 95% confidence level (*α* = 0.05). Our cohort of 66 eyes exceeds this requirement, providing sufficient statistical power to evaluate the architectural ablations and benchmarks presented [7].

Each FAF image has a 30° field of view with pixel dimensions of 768 × 868. All right-eye images were flipped horizontally to maintain consistency, and each sequence was registered to its baseline image. The GA areas on FAF images were graded using the semi-automated software tool RegionFinder 2.6.6.0 (Heidelberg Engineering, Heidelberg, Germany) to delineate areas of atrophy. The FAF data and initial annotations are based on a previous methodology established by Hu et al. [36], with additional longitudinal data acquired for the current study. All annotations were performed at the Doheny Image Reading Center (DIRC) of the Doheny Eye Institute. Each image was initially segmented by a certified reading center grader and subsequently reviewed by a senior grader (A.H.). Discrepancies were resolved and all annotations were finally certified by a senior investigator and DIRC director (S.R.S.).

### 2.2. Hybrid Encoder–Decoder Architecture and Feature Regularization

SWAU-Net utilizes a four-level U-Net backbone with five feature resolutions (L1–L5). At each clinical visit (*t*), the model receives a three-channel input: the FAF image, the current GA lesion mask, and a growth mask representing the expansion since the previous visit.

To maintain high fidelity, the encoder employs a dual-path input design. Because FAF images contain diffuse metabolic signals (lipofuscin noise) while GA masks provide precise geometric boundaries, processing these channels separately ensures that the high-contrast geometric boundaries of the GA masks are not polluted by the diffuse intensity noise inherent in raw FAF imaging. After initial processing, these paths are merged to allow for multimodal reasoning.

Within the encoder, standard residual blocks are augmented with a gated high-frequency Gated Residual Block (GRB) pathway. This modification is specifically designed to protect the junctional zone—the narrow band of tissue where metabolic stress precedes visible atrophy. By using a gated detail pathway, the model avoids the over-smoothing effect common in CNNs, ensuring that the irregular, jagged boundaries of rapid GA expansion are preserved rather than blurred into the background.

To stabilize these features, a Channel-Fusion Bottleneck (CFB) is applied at each resolution. This block, consisting of a 1 × 1, 3 × 3, and 1 × 1 convolutional stack with residual connections, acts as a cross-channel regularizer, forcing the network to align the multimodal information into a shared structural latent space. Finally, to prevent the loss of global anatomical context during downsampling, spatial self-attention is introduced at the deepest levels (L4 and L5). This allows the model to capture long-range interactions across the macula while maintaining geometric coherence.

### 2.3. SWA for Temporal Aggregation

We introduce a Sliding Window Attention (SWA) mechanism designed to serve as the model’s temporal core. Unlike standard global attention, which can be prone to overfitting in low-data regimes, the SWA module explicitly imposes a temporal stationarity prior by applying a single, weight-shared attention operator across multiple shifted temporal windows. This design creates a structural bottleneck that prevents the network from memorizing specific patient visit indices, instead compelling the mechanism to learn a generalized, time-invariant transition function. The shared weights and repeated application over time-shifted inputs act as a crucial form of implicit temporal data augmentation and regularization; by requiring the same parameters to model different segments of the 18-month progression, the network effectively maximizes the signal-to-noise ratio within a limited dataset.

The SWA module aggregates historical spatial features into a unified latent state estimate (*M_t_*), representing the disease state at time *t*. The pipeline consists of:

1. Feature Extraction: The encoder extracts raw spatial features from the FAF and masks.
2. Semantic Alignment: The CFB refines these features into a semantically aligned disease representation ($F_{t}$).
3. Windowed Aggregation: The SWA module applies the weight-shared attention block across a sliding window of three consecutive visits to construct the integrated temporal state.

The SWA module processes 3-frame input tensors sequentially, adding zero-padding to earlier visits to maintain a fixed context window size. Given refined encoder features *F_t_* for time steps *t* = 0 (Month 0), 1 (Month 6), 2 (Month 12), the SWA module cyclically applies its core attention block to construct integrated temporal states *M_t_*:

- $M_{0}=SWA(0,\;0,\;F_{0})$—utilized to predict the state at Month 6.
- $M_{1}=SWA(0,\;F_{0},\;F_{1})$—utilized to predict the state at Month 12.
- $M_{2}=SWA(F_{0},\;F_{1},\;F_{2})$—utilized to predict the state at Month 18.

Within each window, the Transformer performs unmasked self-attention over all tokens. Causality is enforced externally by the windowing mechanism, removing the need for spatiotemporal causal masking. While this sliding window approach is computationally less efficient than standard global attention due to the redundant processing of overlapping frames, it is well-suited for short clinical sequences, where the regularization effect provided by weight-shared windows is critical for achieving generalization.

To maintain global receptive fields with high efficiency, attention is axially factorized into sequential time–width and time–height passes, allowing the model to maintain a global view of the retina while significantly reducing the GPU memory overhead required to process multiple time points simultaneously.

To recover cross-axis spatial dependencies lost during axial factorization, a gated convolutional block fuses information across width, height, and time. This block is integrated via a trainable scalar weight (initialized near zero) to balance global attention with the local anatomy. Finally, macro-residual connections fuse the temporal output with encoder features; deep levels (L4–L5) prioritize high-frequency boundary detail, while shallower levels (L1–L3) enforce temporal smoothness across frames.

### 2.4. State Evolution and Frame Prediction

The architecture is inspired by classical forecasting principles that separate where the disease is now (state estimation) from how it is changing (temporal evolution). The encoder and SWA modules produce a temporally consistent latent state estimate, *M_t_*, while the Dynamics Network (DynNet)—implemented as a 3-level U-Net—predicts the next-step latent state: *E_t+_*_1_ *= DynNet*(*M_t_*).

This hierarchical design allows the network to model multiscale dynamics; the DynNet’s deep layers provide global context to ensure the lesion trajectory remains physically plausible, while its shallow layers capture the subtle, high-frequency shifts in the growth front. By maintaining a separate U-Net for dynamics, the network disentangles spatial representation from temporal forecasting. This separation ensures that imaging noise—such as a dark retinal vessel being mistaken for atrophy—does not propagate into the forecast, as the DynNet evolves abstract disease features rather than raw pixel intensities. Finally, these evolved features undergo channel fusion before the decoder projects them back into image space to generate the next-step prediction, *I_t+_*_1_.

**Figure 2:**
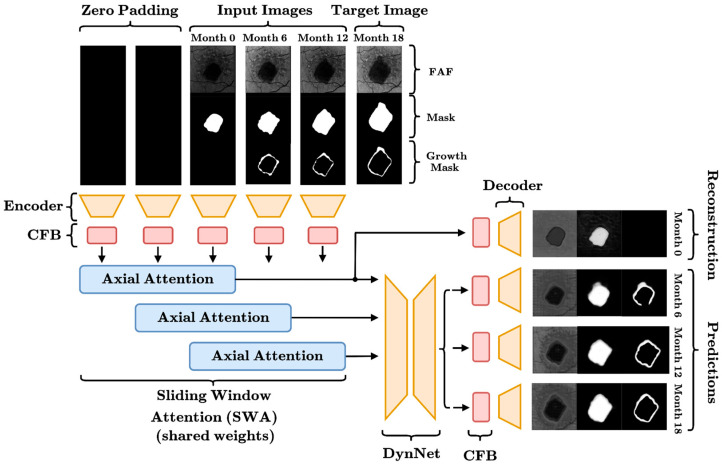
SWAU-Net architecture for longitudinal prediction of GA regions and GA growth. Input images have three channels (FAF, GA Mask, Growth Mask). Each is passed through a U-Net Encoder with spatial attention, followed by the CFB block to encourage richer interactions between channels. A shared self-attention block is applied across three windows in parallel, to enforce a temporal stationarity prior. The architecture employs a multi-task objective: the decoder generates a reconstruction of the initial frame to ensure the SWA module maintains high-fidelity latent representations, while simultaneously generating next-step predictions via the DynNet path. This structure separates the tasks of state estimation (SWA) and prediction (DynNet).

A diagram of the full SWAU-Net architecture is shown in Figure 2.

### 2.5. Synthetic Pretraining via Anisotropic Growth Simulation

To address data scarcity and label sparsity, a synthetic pretraining dataset of 2000 four-frame sequences (8000 images total) was generated to simulate lesion evolution with realistic image noise and spatial irregularities. The simulator outputs sequences containing the FAF images, full lesion masks, and growth masks.

Key fidelity features include:

- Mask Generation: Lesion masks are initialized by thresholding multi-peak Gaussian fields, then expanded through anisotropic directional dilation combined with stochastic erosion/dilation cycles. This mimics the non-uniform growth of clinical GA, where lesions often expand more rapidly in areas of hyperautofluorescence while remaining stable in others. This process generates realistic, jagged growth boundaries that specifically mimic the irregular, directional nature of clinical GA progression, enforcing a high-frequency boundary prior.
- FAF Realism: The simulator incorporates clinical artifacts such as vein-like structures and peripheral noise to ensure the model learns features robust to real-world image interference. This ensures the attention mechanism learns to ignore non-pathological dark structures (like blood vessels) that can mimic the appearance of GA in FAF imaging.

**Figure 3:**
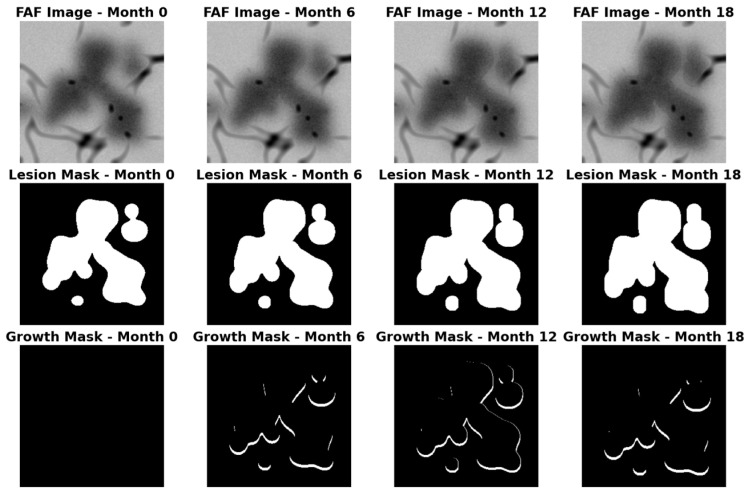
Example synthetic training data. (**Top**) Simulated FAF; (**Center**) GA Mask; (**Bottom**) Growth mask.

Pretraining establishes a strong architectural initialization, enabling faster convergence and enforcing a prior that emphasizes high-frequency boundary fidelity and robustness to noise before fine-tuning on limited clinical data. Example synthetic pretraining data is displayed in Figure 3.

### 2.6. Loss Formulation and Training Strategy

We train the model using a hybrid loss: $L_{Total}\;=\;L_{Pred}\;+\;\lambda_{Recon}\;\cdot \;L_{Recon}$, with $\lambda_{Recon}<0.5$, balancing prediction and reconstruction objectives. The prediction loss $L_{Pred}$ is applied to frames *I*_1_, *I*_2_, and *I*_3_ and combines a Soft Dice Loss (SDL) on the GA mask and growth mask—weighted heavily on the sparse growth regions—with a small Binary Cross-Entropy (BCE) term for stability, and an L1 loss on the FAF channel to ensure accurate reconstruction.

The model is trained end-to-end on the synthetic dataset to establish strong priors for spatial feature recognition and boundary localization. The model is then fine-tuned on real clinical sequences. We use gradient accumulation to simulate larger batch sizes to reduce variance in small datasets. Each batch of images is augmented online (during training), applying geometric transformations (rotation, flip, zoom) and targeted random intensity corruption of the FAF channel. This ensures that the network sees a different augmented version of each image every epoch, encouraging it to prioritize stable geometric features over noisy intensity cues and improving generalization in low-data settings.

## 3. Experiments

### 3.1. Experimental Setup

The SWAU-Net model uses a U-Net with four down-sampling stages, producing 16 × 16 bottleneck features from a 256 × 256 input (downsampled from the original 768 × 868). This resolution was selected to balance spatial detail with the memory requirements of training longitudinal sequences. With a base channel width of *C* = 16, the deepest layer contains 256 channels. The SWA block operates on feature maps up to 32 × 32 × 128 at level L4 and 16 × 16 × 256 at level L5. The resulting 16 × 16 bottleneck allows for efficient Transformer-based temporal modeling while the preceding convolutional stages retain sufficient resolution to resolve the geometric frontiers of GA expansion. The model contains approximately 8.3 million parameters. All ablated models are built upon this shared backbone, utilizing the same dual-path input, Gated Residual Blocks (GRBs), and two-stage training (synthetic pretraining followed by clinical fine-tuning), unless explicitly ablated below.

Due to the limited sample size (*n* = 66), a five-fold cross-validation strategy was employed, with four folds containing 13 eyes each and one fold containing 14 eyes. For each iteration, four folds were used for training and one fold was reserved for testing, ensuring that every eye in the dataset was included in the test set exactly once.

To validate the efficacy of our design, particularly the regularization and decomposition scheme required for the low-data regime, we designed the following ablations and benchmarks (Table 1):

These ablations are designed to isolate which components are necessary for stable prediction in the low-data regime. Removing spatial attention tests whether non-local pixel interactions matter beyond convolutional context, while removing the CFB measures how much the model relies on explicit fusion of the three input modalities. The three SWA ablations progressively test (1) whether temporal weight-sharing is required to regularize the Transformer, (2) whether SWA’s adaptive, non-local temporal reasoning is doing more than a simple causal convolutional mixer, and (3) whether our decoupled CNN → Attention → DynNet design offers advantages over a fully recurrent ConvLSTM, which is sequential and cannot leverage the parallel computation of SWAU-Net. The DynNet ablation tests whether explicitly separating state estimation and prediction is necessary to prevent transient imaging noise from corrupting the longitudinal forecasting task. Finally, we evaluate if synthetic pretraining and data augmentation are essential for stabilizing the attention layers when training on small clinical datasets.

We pretrain all models for 50 epochs on the synthetic dataset, then finetune on real data for an additional 60 epochs. Optimization is performed using the Adam optimizer with a learning rate of 1 × 10^−3^ during pretraining and 1 × 10^−4^ during finetuning. We use a Dropout rate of 0.2.

### 3.2. Results

**Figure 4:**
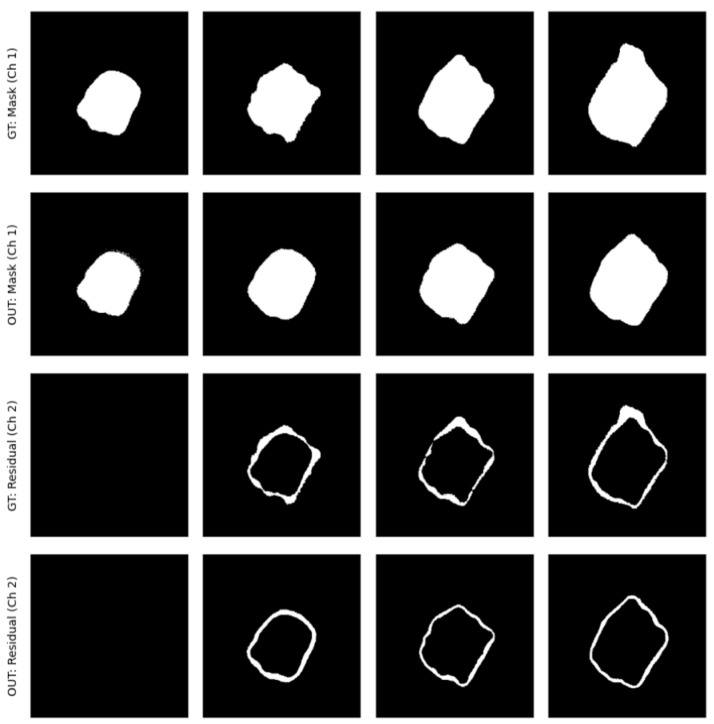
Ground truth masks and predictions for Months 0, 6, 12, 18 (left to right). (**First row**) Ground truth GA masks. (**Second row**) Predicted masks. (**Third row**) Ground truth growth masks. (**Fourth row**) Predicted growth masks.

Figure 4 compares the ground truth GA masks and growth regions with the predictions generated by the trained SWAU-Net model.

We evaluated model performance by computing the Dice Similarity Coefficient (DSC) between the predicted and ground-truth GA masks, as well as the corresponding growth masks. We performed five-fold cross validation, using the median DSC for each test fold, and averaging this over the last 10 epochs of training for additional stability (epochs 50–60). Results are displayed in Table 2. We evaluated statistical significance using a corrected paired *t*-test (Nadeau and Bengio), which accounts for the correlation of train/test folds in *k*-fold cross-validation, as shown in Table 2. To maintain statistical rigor across the nine architectural ablations and benchmarks, a Bonferroni correction was applied to the analysis of the Growth Mask DSC. This resulted in a conservative significance threshold of *α* = 0.0056 (0.05/9). These results are shown in Table 3. Note that metrics for the growth mask are more informative than those for the total GA mask, since even minor errors in the narrow growth region can substantially affect lesion expansion predictions.

SWAU-Net achieved a median Mask DSC of 0.94 ± 0.01 and Growth Mask DSC of 0.66 ± 0.01. After applying a Bonferroni correction (*α* = 0.0056), the most critical components were confirmed to be those enforcing stability in the low-data regime: removing the Sliding Window Attention (SWA) weight-sharing (SWA Ablation 1, *p* = 0.0002) and the Channel Fusion Bottleneck (CFB) (*p* = 0.0005) caused the greatest performance collapse. This confirms that temporal stationarity and multimodal fusion are the primary drivers of model robustness.

Ablation 1, the primary test of the SWA mechanism’s efficacy, showed a severe decline in performance on the growth prediction tasks (Growth Mask DSC of 0.52 ± 0.02), demonstrating the necessity of the temporal stationarity prior in data-scarce settings. While the model showed improved performance trends through non-local reasoning over a simple convolutional mixer (SWA Ablation 2, *p* = 0.0116) and through Synthetic Pretraining (*p* = 0.0109), these comparisons did not meet the strict significance threshold following Bonferroni adjustment.

Finally, SWAU-Net achieved generalization performance comparable to the best recurrent model (SWA Ablation 3, *p* = 0.9899), validating our central thesis: by injecting strong temporal priors into a Transformer framework, SWAU-Net stabilizes the expressive attention mechanism to achieve recurrent-level robustness without sacrificing parallel computation.

## 4. Discussion and Conclusions

We introduced SWAU-Net, a hybrid CNN–Transformer architecture that embeds temporal and spatial consistency priors for robust GA progression forecasting. While previous CNN-RNN approaches such as ReconNet [37] successfully demonstrated the utility of recursive modeling for GA, our results (Table 2) suggest that SWAU-Net provides a more stable alternative for resolving high-frequency growth boundaries. SWAU-Net achieves a DSC of 0.66, demonstrating superior preservation of growth boundaries compared to recursive baselines, which are often prone to temporal over-smoothing.

SWAU-Net offers a more robust alternative to unregularized Transformer architectures. While global attention mechanisms provide high expressivity, they frequently overfit on limited clinical datasets by attempting to model spurious long-range correlations. In contrast, SWAU-Net utilizes a weight-shared SWA core that imposes a structural bottleneck, forcing the model to learn a time-invariant transition function. This regularization ensures that the model captures meaningful longitudinal trends rather than patient-specific noise, providing a superior inductive bias for the low-data regime compared to high-parameter global or hierarchical Transformers.

Furthermore, while 3D structural models such as Deep-GA-Net [31] have advanced GA prediction by utilizing Optical Coherence Tomography (OCT) to identify sub-retinal biomarkers, SWAU-Net demonstrates that optimized 2D architectures can achieve high precision (DSC 0.66) on Fundus Autofluorescence (FAF) imaging alone. This is particularly relevant for clinical trial settings where FAF remains a primary endpoint for evaluating lesion expansion.

In a clinical context, this enhanced stability translates directly to a more reliable estimate of lesion expansion volume and timing, enabling clinicians to optimize patient monitoring schedules and better time emerging interventional therapies that rely on predicting the boundary of future atrophy. By accurately forecasting the atrophic frontier, SWAU-Net facilitates a shift from reactive monitoring to proactive, personalized care. These trajectories allow for optimized follow-up schedules—reducing the burden on slow-progressing patients while identifying patients with fast progression for early intervention. Moreover, in the context of emerging complement-inhibitors, such modeling serves as a vital tool for clinical trial design, providing a stabilized natural history baseline to more precisely measure therapeutic efficacy.

A limitation of this study is the reliance on discrete, fixed-interval time steps. In clinical practice, patient follow-ups are often irregularly spaced, which the current fixed-window approach does not explicitly model. Future efforts will investigate Stochastic Differential Equations (SDEs) and Neural Ordinary Differential Equations (N-ODEs) to transition from discrete sequences to continuous-time modeling, allowing for more flexible handling of irregularly spaced clinical visits.

A further limitation is the 18-month follow-up period, which may be insufficient to fully capture long-term growth dynamics. Clinically, foveal lesions are known to develop more slowly than extrafoveal lesions and exhibit a ‘foveal-sparing’ tendency, growing more rapidly toward the periphery than toward the center [47]. Because our 18-month window may catch lesions in different phases of this directional growth, future studies with longer longitudinal tracking (e.g., 3–5 years) are necessary to validate the model’s ability to predict these complex, non-isotropic expansion patterns.

While this study demonstrates the effectiveness of SWAU-Net in capturing temporal trends, we did not explicitly categorize baseline FAF patterns (e.g., focal, banded, or diffuse) according to standard clinical classifications. While the model’s spatial attention mechanism is designed to learn these features from the raw imaging data, incorporating explicit clinical phenotypes as auxiliary inputs might further improve forecasting accuracy in the low-data regime.

While FAF imaging provides a high-contrast representation of RPE health, GA is fundamentally a three-dimensional pathological process involving the progressive loss of the photoreceptors, RPE, and choriocapillaris. Future iterations of the SWAU-Net could benefit from integrating 3D structural data from Optical Coherence Tomography (OCT), which has been shown to provide complementary information regarding sub-structural changes such as reticular pseudodrusen or shallow pigment epithelial detachments that may precede RPE loss [48].

Furthermore, while this study focuses on anatomical growth, incorporating functional endpoints such as macular microperimetry would provide a more comprehensive assessment of the clinical impact of predicted expansion [49,50]. Finally, as this study was conducted on a single-center dataset of 66 eyes, external validation in larger, multi-center cohorts is necessary to confirm the generalizability of the SWAU-Net architecture across different imaging devices and more diverse patient populations.

In summary, SWAU-Net combines the inductive strengths of CNNs with the adaptive context of Transformers to provide a stable, high-performance tool for GA forecasting. This architecture offers a robust foundation for enhancing clinical trial design and personalizing long-term patient monitoring of GA in AMD. Beyond GA, this architecture holds substantial promise for a wide range of applications, including disease trajectory modeling in other progressive ophthalmic diseases.

## Figures and Tables

**Table 1:** Ablations and benchmarks.

Model/ Ablation Name	Core Modification	Primary Hypothesis Tested
No Spatial Attention	Removes all Spatial Self-Attention layers within the Encoder and DynNet.	Tests the contribution of non-local pixel interactions vs. purely local convolutional processing in maintaining feature fidelity.
No Channel Fusion Bottleneck	Replaces all Channel Fusion Bottleneck (CFB) blocks with simple residual skips and concatenation.	Tests the importance of explicit semantic alignment of multi-modal input features (FAF, GA Mask, Growth Mask).
SWA Ablation 1 (Standard Attention)	Replaces SWA with Standard Causal Axial Attention (non–weight-shared).	Tests whether SWA’s temporal-stationarity prior (via weight-sharing) is needed to prevent highly expressive but unregularized Transformers from overfitting small datasets.
SWA Ablation 2 (Temporal Aggregator)	Replaces SWA with a simple convolutional aggregator (feature concatenation) at L1–L3.	Tests whether the stable CNN backbone (DynNet-based decomposition) alone is sufficient, or if explicit attention-based temporal aggregation is required.
SWA Ablation 3 (ConvLSTM)	Replaces the entire SWA core with a sequence of standard ConvLSTM cells, but retains spatial attention and CFB.	Tests whether our decoupled hybrid architecture (CNN → Attention → DynNet) provides stability or expressivity benefits over conventional coupled ConvLSTM approaches.
No DynNet	Removes the Dynamics Network (DynNet) and reconstruction loss, and predicts directly from the estimated state.	Tests whether the explicit separation of state estimation and temporal evolution acts as a regularizer against the entanglement of spatial noise and temporal forecasting.
No Synthetic Pretraining	Skips phase 1 of training and initializes the model directly on the small clinical dataset.	Tests whether establishing a strong, generalized prior (especially for high-frequency boundaries) is required for Transformer components to converge effectively in the target domain.
No Data Augmentation	Removes online data augmentation, including FAF intensity jitter and noise, and geometric transformations (flips, rotations, etc.).	Tests whether online data augmentation is necessary to stabilize attention-based layers on the small clinical dataset.

**Table 2:** Five-fold validation accuracy for GA region mask DSC and growth mask DSC for SWAU Net and ablations/benchmarks. Plus/minus signs indicate one standard deviation. Note: We indicate robust statistical significance over the ablated model after Bonferroni correction with (*) (see Table 3).

Model	Mask DSC (Mean ± SD)	Growth Mask DSC (Mean ± SD)
SWAU Net	0.94 ± 0.01	0.66 ± 0.01
Spatial Attention Ablation	0.94 ± 0.01	0.64 ± 0.01
CFB Ablation *	0.92 ± 0.01	0.53 ± 0.02
SWA Ablation 1 (Standard Attention) *	0.92 ± 0.02	0.52 ± 0.02
SWA Ablation 2 (Temporal Aggregator)	0.94 ± 0.01	0.63 ± 0.01
SWA Ablation 3 (Attention-ConvLSTM)	0.94 ± 0.01	0.66 ± 0.01
DynNet Ablation	0.94 ± 0.01	0.63 ± 0.03
No Synthetic Pretraining	0.94 ± 0.01	0.64 ± 0.02
No Data Augmentation	0.94 ± 0.02	0.63 ± 0.04

**Table 3:** Corrected Paired *t*-test (Nadeau and Bengio).

Model	*p*-Value
SWAU Net vs. Spatial Attention Ablation	0.0254
SWAU Net vs. CFB Ablation	0.0005
SWAU Net vs. SWA Ablation 1 (Standard Attention)	0.0002
SWAU Net vs. SWA Ablation 2 (Temporal Aggregator)	0.0116
SWAU Net vs. SWA Ablation 3 (Attention-ConvLSTM)	0.9899
SWAU Net vs. DynNet Ablation	0.1187
SWAU Net vs. No Synthetic Pretraining	0.0109
SWAU Net vs. No Augmentation	0.1415

## Data Availability

The datasets and code generated during this study are accessible from the corresponding author based on reasonable request and subject to the regulations of the institute.
